# Exploring Non-Invasive Salivary Biomarkers for Acute Pain Diagnostics: A Comprehensive Review

**DOI:** 10.3390/diagnostics13111929

**Published:** 2023-05-31

**Authors:** Laura Stendelyte, Mantas Malinauskas, Dovile Evalda Grinkeviciute, Lina Jankauskaite

**Affiliations:** 1 Faculty of Medicine, Lithuanian University of Health Sciences, LT-44307 Kaunas, Lithuania; 2 Institute of Physiology and Pharmacology, Medical Academy, Lithuanian University of Health Sciences, LT-50161 Kaunas, Lithuania; 3 Department of Pediatrics, Medical Academy, Lithuanian University of Health Sciences, LT-50161 Kaunas, Lithuania

**Keywords:** acute pain, biomarker, non-invasive, salivary diagnostics, pediatric

## Abstract

Pain is one of the most common complaints leading to a pediatric emergency department visit and is associated with various painful procedures, leading to increased anxiety and stress. Assessing and treating pain in children can be challenging, so it is crucial to investigate new methods for pain diagnosis. The review aims to summarize the literature on non-invasive salivary biomarkers, such as proteins and hormones, for pain assessment in urgent pediatric care settings. Eligible studies were those that included novel protein and hormone biomarkers in acute pain diagnostics and were not older than 10 years. Chronic pain studies were excluded. Further, articles were divided into two groups: studies in adults and studies in children (<18 years). The following characteristics were extracted and summarized: study author, enrollment date, study location, patient age, study type, number of cases and groups, as well as tested biomarkers. Salivary biomarkers, such as cortisol, salivary α-amylase, and immunoglobulins, among others, could be appropriate for children as saliva collection is painless. However, hormonal levels can differ among children in different developmental stages and with various health conditions, with no predetermined levels of saliva. Thus, further exploration of biomarkers in pain diagnostics is still necessary.

## 1. Introduction

Pain is one of the most common complaints for referral to the pediatric emergency department (PED). It occurs as a result of different traumas and injuries or infectious or inflammatory diseases. Moreover, children in PED often undergo painful procedures such as venipuncture, urine catheterization, or casting, which can cause heightened fear and somatization in children and increased anxiety in parents. According to the International Association for the Study of Pain (IAPS), pain is defined as “an unpleasant sensory and emotional experience associated with actual or potential tissue damage” [1,2]. Evaluating and treating pain in children, particularly in the PED, can pose challenges. This is due to the unique nature of pain in pediatric patients, which requires consideration of various factors such as cause, nature, age, developmental stage, communication abilities, pain perception, and past medical experiences of the child. All of these aspects should be carefully evaluated and addressed [3]. Pain in children is often underdiagnosed, and the severity of their pain may not be accurately determined, resulting in inadequate pain management [4]. The inaccurate assessment of pain level is due to the pathophysiological mechanisms of pain in childhood. In the past, there were many misconceptions about pain and the neurobiology of pain in children, such as the belief that “children do not comprehend pain”, “newborns do not feel pain and therefore do not require anesthesia”, or “girls exaggerate their pain, while boys are expected to tolerate ’’ [5,6,7,8]. However, recent studies have shed light on the importance of appropriately addressing and managing pain in children.

The provision of adequate pain relief treatment for children is hindered by various factors, including the medical staff’s perception of pain, fear of using pharmacological agents, and a lack of knowledge and experience in selecting the appropriate method of analgesia hinder the provision of appropriate pain relief treatment for children [9,10,11,12,13,14]. Nevertheless, there is currently a growing emphasis on patient safety and evidence-based practices, which has increased the relevance of addressing pain in children [11,15,16,17]. Inadequate analgesia can have negative effects on a child’s development, including impaired sleep, anxiety, stress, and negative memories associated with healthcare facilities and services [18]. Despite the availability of various pain assessment tools, there is no universally accepted method for assessing pain in children in different healthcare settings [19]. The choice of acute pain evaluation method depends on various factors, including the healthcare provider’s experience and individual understanding of pain, the child’s age, development, existing pathology, state of consciousness, cultural background, and sometimes the attitude of the institution itself or the national medical system [20,21]. Currently, clinical pain assessment relies mostly on self-reported measures, such as numerical pain ratings or face scales, which are valid and easy to use but may not fully convey the complexity of the pain experience. Physiological parameters such as heart rate (HR), respiratory rate (RR), arterial blood pressure (ABP), and others are included in pain evaluation [22]. However, there are currently no clinically applicable and confirmed objective and non-invasive biomarkers from saliva or sweat to accurately determine the pain level and adequacy of pain relief, making the perception of pain entirely subjective [23]. The objective assessment of pain is essential for achieving optimal pain estimation and reduction. The use of specific biomarkers such as proteins and hormones in non-invasive salivary and sweat biomarkers is a relatively new area of research that could provide more objective pain assessment and contribute to reduced fear and anxiety in pediatric patients. Thus, our objective was to review and summarize the literature on different non-invasive biomarkers, including proteins and hormones present in saliva and sweat, for pain assessment. We included both pediatric and adult studies in our analysis to evaluate existing and potential biomarkers in pediatric acute pain diagnostics. The implementation of these biomarkers in clinical practice could lead to a more objective pain assessment and contribute to reduced fear and anxiety in pediatric patients. Moreover, it could result in more individualized pain relief and better clinical outcomes.

## 2. Materials and Methods

### 2.1. Eligibility Criteria

Studies including different novel protein and hormone biomarkers in acute pain diagnostics and not older than 10 years were eligible to be included in this review (Table 1).

### 2.2. Inclusion and Exclusion Criteria

We excluded studies that focused on chronic pain as well as those emphasizing physiological parameters such as RR, HR, ABP, and others or used serum, plasma, and urine proteins and hormones. In vitro or animal studies were also excluded. Furthermore, literature reviews, systematic reviews, and meta-analyses were not included in our analysis. The specific inclusion criteria are represented in Table 2.

### 2.3. Search Strategy

The literature was identified by two independent reviewers. The search has been performed in Medline (Pubmed) and Google Scholar until 1 February 2023. The search strategy included a combination of the following terms: “acute pain” AND “assessment” OR “evaluation” OR “diagnostics” AND “marker” OR “biomarker” OR “protein” OR “hormone” OR “cytokine” OR “interleukin” AND “saliva” OR “sweat”. 

### 2.4. Data Extraction

Following the primary study collection, all articles were divided into two groups: studies in adults and studies in children (<18 years). Further, all the study characteristics were extracted and summarized as follows: the author of the study, enrollment date, study location, patient age, study type, number of cases and groups, as well as tested biomarkers. Moreover, the main findings were further summarized in the conclusion and notes (Table 3).

## 3. Results

The search returned 36,180 records up to the 1st of February 2023. After activating filters, we selected 763 articles. After duplicate removal, the titles and abstracts were manually sorted and matched according to the inclusion and exclusion criteria. A total of 55 full-text articles were further evaluated. Finally, 30 were included in our final analysis, as follows: 10 studies, including children, and 20 adult studies. Afterward, details were extracted for each article as in the methods. The information regarding the articles included in the study is summarized in Table 3.

## 4. Discussion

### 4.1. The Most Commonly Analyzed Biomarkers

#### 4.1.1. Cortisol

Cortisol, which is produced by the adrenal gland in response to stress, is a hormone that can be measured in various bodily fluids. It is one of the most extensively studied salivary markers in acute pain, but the data on its use are highly varied due to its reputation as the “stress hormone”. Despite this, it is frequently evaluated in adults since salivary cortisol values for different conditions are proportionate to serum levels [51,52]. Salivary cortisol is advantageous as a biomarker in acute pain measurement because it is non-invasive, easy to collect, and can be measured in real-time. Studies suggest that the cortisol awakening response may be a marker for stress sensitivity and/or the anticipation of impending stress [34]. In a study conducted in South Korea with 46 healthy male participants, researchers observed that stress increases the activities of the hypothalamic (H)–pituitary (P)–adrenal (A) axis and decreases HP gonadal axis activity, contributing to increased pain perception [35]. Additionally, cortisol levels and pain ratings were significantly higher under anxiety than under rest, suggesting that cortisol may influence pain responses and that managing anxiety related to the elevation of cortisol may relieve acute clinical pain [36]. In the study of 99 female twin pairs analyzing salivary cortisol, researchers observed that the HPA axis was related to pain sensitivity and endogenous pain inhibition in response to the negative feedback paradigm and in recovery periods, indicating that cortisol can be a useful biomarker of pain sensitivity [37]. However, in a few studies, the role of cortisol as a specific and sensitive biomarker in acute pain diagnostics or even its role in stress has been controversial [38,39]. For example, a study by Sadi et al. failed to show any significant correlation between dental procedure-induced anxiety and cortisol [38]. Blum et al. observed that cortisol and copeptin were not useful biomarkers for acute to moderate pain. Therefore, it may not be necessary to consider acute moderate pain as a confounding factor for cortisol or copeptin levels in clinical settings [18].

Looking for less invasive estimates, salivary markers including cortisol would be very reasonable in the pediatric population [24]. Yet, to date, there is no clear scale of salivary cortisol normal values in children of different ages and conditions; thus, it is still complicated to define it in the sense of pain diagnostics. Acute pain diagnostics is more complex, especially when each child’s reference values (diurnal fluctuations, value before painful stimulus) are not known. Despite the heterogeneous data regarding cortisol as a biomarker for acute pain in children, its potential has been demonstrated in several studies. Brummelte et al. observed that total diurnal cortisol was associated with neonatal pain-related stress and CBCL/6-18 (child behavior checklist) scores, particularly in boys [24]. Stoye et al. found higher salivary cortisol levels in preterm infants 30 minutes after a stressor (*p* = 0.009), but not before (*p* = 0.09) or 20 min after (*p* = 0.10) [31]. Additionally, Brockington et al. suggested that a combination of increased oxytocin and reduced cortisol may reduce negative emotions and pain perception in children during hospitalization [25]. However, a study on orthodontic tooth procedures did not result in significant alterations in salivary cortisol levels [26].

#### 4.1.2. Salivary α-Amylase

The biomarker salivary α-amylase (sAA) has been extensively studied in relation to pain, but recent studies in the past decade have not found it to be a promising pain biomarker [28,40]. However, a study by Jenkins et al., (2018) involving 73 tumor patients in the USA found that sAA increased immediately after a painful stimulus. This suggests that sAA measurements should be taken during and after painful procedures [27]. Portable stress measurement devices, such as the sAA biosensor, have the potential to monitor underlying psychological differences and predict a patient’s postoperative response, ultimately resulting in better pain control in surgical patients and, thus, improving patient care and satisfaction [40]. Nevertheless, it should be noted that the usefulness of sAA as a pain biomarker remains controversial. While some studies have shown promising results, others have failed to demonstrate a significant correlation between sAA and pain or stress. For example, a study of experimentally induced pain did not find a significant influence of acute pain on the levels of sAA [41]. However, this study used a short-lasting, high-intensity pain model, which may have resulted in a pain state that was too short-lasting to activate the autonomic nervous system (ANS), leading to sAA secretion [41]. Furthermore, orthodontic tooth removal did not cause significant changes in sAA in response to pain and stress in children [26]. Similarly, a study by Sadi et al. failed to show any significant correlation between dental anxiety and sAA values [38]. However, some studies have suggested that sAA may be useful as a biomarker in certain situations, such as during and after painful procedures. For instance, a study of venipuncture-induced stress found that sAA increased in response to the acute stress and remained elevated 15 min after the procedure [42].

#### 4.1.3. Salivary Immunoglobulin A and Other Immunoglobulins

Salivary immunoglobulin A (sIgA) is the main class of antibodies found in the body’s secretory fluids, including saliva [53]. Researchers have investigated sIgA as a potential biomarker for pain diagnostics. In children undergoing orthodontic treatment, a negative correlation was observed between oral pain intensity and sIgA levels. This may indicate that sIgA may play a protective role during orthodontic treatment, reducing the pain experienced by patients [24]. Additionally, studies by Sobas et al. found that sIgA concentrations were significantly higher after surgery at each visit compared to the baseline values (*p* = 0.053, *p* < 0.001, respectively) [43]. Another study by Sobas et al., including 34 subjects, found that sIgA showed acceptable levels of reproducibility and could be used as a potential salivary biomarker in pain diagnostics [39]. A study by Marques-Feixa et al. suggested that puberty may impact sIgA in response to acute stress stimulation [33]. In a case-control study, sIgA was found to complement pain assessment and reinforce the suitability of the PAINAD (pain assessment in advanced dementia) scale for evaluating pain [54]. However, a study by Bialka et al. did not find any significant differences in sIgA levels between 6 and 24 h after surgery, along with other biomolecules such as testosterone, cortisol, sAA, and β-endorphin [45].

### 4.2. Other Biomarkers

#### 4.2.1. Oxytocin

The neuropeptide oxytocin (OXT), produced in the hypothalamus, is extensively studied for its anti-stress-like and anxiolytic-like effects [55]. OXT is also believed to play a significant role in pain modulation. Recent research has revealed its potential for mitigating the effects of chronic stress on the developing brain [56]. In a case-control study of 21 preterm infants, Filippa et al. investigated whether the mother’s voice could provide effective and safe analgesia for preterm infants and whether endogenous OXT levels were related to pain response [29]. Their findings suggest that the OXT system plays a crucial role in restoring and reconstructing the infant’s resilience in response to painful stimuli [46]. Moreover, an increase in OXT levels combined with a decrease in cortisol levels may reduce the negative emotions associated with hospitalization and influence children’s perception of pain [25]. However, studies in adults have not shown any significant influence of either conditioned endogenous OXT release or exogenous OXT administration on pain sensitivity [46].

#### 4.2.2. Testosterone

Testosterone has been identified as a promising marker that may be linked to pain regulation [35]. Research suggests that acute clinical pain may be relieved by managing stress and controlling stress-related testosterone levels. Studies have shown that salivary testosterone levels are significantly lower during stressful conditions compared to when at rest [35]. However, Sobas et al. found no association between testosterone and pain in healthy individuals [39]. Further, their study revealed that anxiety was associated with lower testosterone levels, suggesting that managing anxiety may be an effective way to alleviate acute clinical pain by reducing testosterone levels [36].

#### 4.2.3. Soluble Tumor Necrosis Factor-Alpha Receptor

Tumor necrosis factor alpha (TNFα) is a pro-inflammatory cytokine, while soluble tumor necrosis factor-alpha receptor-II (sTNFαRII) reflects its activity. According to Sobas et al.’s research, sTNFαRII has the potential to serve as a diagnostic marker for pain in healthy individuals [39]. Furthermore, in another study by the same author, sTNFαRII levels post-surgery were significantly higher at each follow-up visit compared to the baseline values (*p* = 0.053, *p* < 0.001, respectively) [43]. However, cortisol awakening response (CAR) and sTNFαRII showed no association with acute pain [34].

#### 4.2.4. Chemokines and Cytokines (IL-1β, IL8 and Others)

Chemokines and cytokines have also been proposed to be linked to pain. Elevated levels of interleukins such as IL-1α and IL8 indicate continuous immune system upregulation among individuals experiencing pain [47]. Carlton et al. observed that salivary levels of interleukin (IL-1β, IL-4) were elevated in patients with burn characteristics compared to the healthy controls [32]. In a case-control study by Singh et al., 16 patients undergoing canine tooth removal were included and tested for IL-1β at 24 h and after 2 months after the procedure [48]. The authors found significantly higher levels of IL-1β levels at 24 h and after 2 months of the initial canine tooth movement compared with the control teeth. However, these markers and all the interleukins are challenging to analyze and relate solely to a painful stimulus as their changes can be affected by various inflammatory conditions, such as inflammatory dental diseases or the presence of dental plaque [48].

#### 4.2.5. Opiorphin

Opiorphin is a naturally occurring human peptide that protects enkephalins by inhibiting the enkephalin-degrading enzymes and prolonging their effects. Inflammation-related dental pain was found to increase salivary opiorphin levels [44], which were strongly correlated with the reported pain level. Moreover, the opiorphin values were affected by the extent of the inflammation, whether pulpal or periodontal [44].

### 4.3. Biomarkers to Be Considered in Pain Diagnostics

#### 4.3.1. Nerve Growth Factor

Various studies have identified nerve growth factor (NGF) as a potential salivary biomarker in pain diagnostics. As a neuropeptide, NGF modulates the expression of peripheral and central pain-related markers [49,57]. Moreover, inflammation can lead to adjacent nociceptive neuron sensitization via NGF. Most studies on NGF have focused on chronic pain or exacerbations of chronic diseases [57]. Elevated levels of NGF have been observed in the saliva, general circulation, and synovial fluid of patients with different pain conditions [58]. However, there is a lack of studies analyzing NGF’s role in acute pain conditions (e.g., trauma, acute visceral pain, etc.) [49]. Some studies have shown NGF’s role in hyperalgesia during acute experimental pain [59]. In addition, local NGF levels increase within a few hours after acute injury and inflammation [60]. Despite this, using NGF as a salivary biomarker in acute pain diagnostics may present diagnostic challenges, particularly in children, where saliva sampling can be complicated, and reference values of NGF have not yet been determined.

#### 4.3.2. Substance P

Another interesting candidate for acute pain diagnostics could be salivary substance P (sP). It is released in response to noxious stimuli and is present in both the central and peripheral nerves [30]. However, the data on sP during acute pain are limited, and most studies have analyzed sP in the blood (serum, plasma). The main challenge with salivary sP, similar to NGF, is the method of saliva sampling. In a study by Jasim et al., sP levels showed large variations between the five different saliva collection methods. Post-hoc analysis revealed significantly higher levels of sP in saliva derived mainly from the sublingual and submandibular glands, such as unstimulated whole saliva (235 ± 137 pg/mL), unstimulated sublingual saliva (257 ± 89 pg/mL), and stimulated sublingual saliva (370 ± 185 pg/mL), when compared to saliva with high parotid content, e.g., whole stimulated saliva (23 ± 27 pg/mL) and parotid saliva (11 ± 17 pg/mL) [49]. However, another study by the same author found that sP expression in unstimulated whole saliva and plasma did not significantly change over time, and the peptide was not detectable in all samples of stimulated whole saliva, making accurate analysis difficult [50].

#### 4.3.3. Glutamate, Brain-Derived Neurotropic Factor

There is limited research on the potential of such markers as glutamate or brain-derived neurotropic factor (BDNF) for acute pain diagnostics. Glutamate, a key excitatory neurotransmitter in the nervous system, is involved in neuronal activation and mediates synaptic transmission of sensations such as pain. Its levels are highest in the early morning and decrease throughout the day [50]. However, two studies by Jasim et al. found no significant changes in salivary glutamate levels across the day [49,50]. BDNF is another neuropeptide that plays an important role in the development of pain and hyperalgesia. Jasmin et al. used western blot-based technology to detect BDNF isoforms in saliva and found a difference in BDNF levels throughout the day and between unstimulated and stimulated saliva [49,50].

#### 4.3.4. Other Biomolecules

Such biomarkers as somatostatin, neuropeptide Y, dynorphin A (DA), serotonin, chromogranin A (CgA), and prolactin, among others, are being studied for their potential use in pain diagnostics in experimental settings [26,47,49]. Somatostatin and neuropeptide Y are involved in the pain regulatory pathway, and Symons et al. found that their levels were higher in children with pain than in those without pain [47]. Dynorphin is a neuropeptide that binds to the kappa opioid receptor (KOP), which is part of the G protein-coupled receptor family and is involved in pain regulation. DA has also been shown to be important in distinguishing between the “pain” and “no pain” groups. While the separation was not as clear for the metabolites, valine, proline, hypoxanthine, propionate, formate, and acetate were among the most important metabolites for partially distinguishing the “pain” group from the “no pain” group [47]. CgA, a protein found in neuroendocrine cells, could be a potential candidate for pain markers, but Bavbek et al. did not observe significant differences in CgA concentrations [26]. Calcitonin gene-related peptide (CGRP) is involved in nociceptive pathways in the peripheral and central nervous systems, and its receptors are expressed in pain pathways. Total CGRP expression was significantly higher in all stimulated saliva samples, whether chemically or mechanically stimulated, compared to unstimulated saliva samples [49].

### 4.4. Could It Be Translated into Pediatric Acute Pain Diagnostics?

Currently, pain diagnosis relies on various pain scales and physiological parameters, such as HR, RR, or ABP [59]. However, this approach may not always be sensitive and specific enough for pediatric populations, especially neonates, non-verbal children, and children with disabilities. Therefore, various studies have investigated the use of different blood and saliva biomarkers for objective pain evaluation. In our review, we observed that the majority of the studies were performed on an adult population [34,35,36,37,38,39,40,41,42,43,45,46,47,48,49,50]. In addition, it is easy to explain that biomarkers, e.g., cortisol, are already measured in the blood with pre-defined reference values. Thus, salivary reference levels could be easily calculated. Meanwhile, children are a heterogeneous population in different stages of development, with different hormonal levels and no clear predetermined levels. Furthermore, the studies have used different time points, i.e., some of the studies focused on morning levels, others evaluated before and after painful stimulus, and some other studies did monitor biomarker changes longitudinally [24,26,41,46]. The inconsistency of the results, time points, measurement methods, and sample collection methods makes it difficult to summarize and include those biomarkers in pediatric pain evaluation. Nevertheless, salivary biomarkers are suitable for children because they are easy and painless to collect, can be evaluated in real-time, and enable repeated measurements. However, saliva collection could be challenging under specific conditions, such as dehydration, and biomarkers could give false-positive or false-negative values due to certain conditions such as the use of glucocorticoids, previous food intake, anxiety, or acute inflammation [47,48,49]. Furthermore, current protein and hormone level measurements via ELISA, Western blot, or mass spectrometry are not clinically relevant in the context of acute pain diagnostics, and only a few studies included on-site diagnostic devices. Therefore, more studies must be performed to develop on-site diagnostic devices in the context of acute pain [61]. Finally, sensitivity and specificity could be increased with a combination of a few biomarkers or by adding biomarker results to pain scales and physiological parameters. In conclusion, the field of biomarkers in pain diagnostics is still to be explored further regarding higher clinical adaptability, efficacy, and evidence-based results.

### 4.5. Study Limitations

Our review has certain limitations. Firstly, we limited our search to publications within the last 10 years, which may have resulted in missing some important publications. Additionally, we excluded animal studies, potentially missing the discovery of new proteins. Our analysis did not include “gray-literature”. Furthermore, we did not include studies related to chronic pain or pain exacerbation, which may have prevented the discussion of certain hormones and proteins. Despite its limitations, we believe that our review will be valuable to the field of non-invasive pain diagnostics, particularly for pediatric patients, since there is a shortage of information on non-invasive diagnostic biomarkers for acute pain.

## 5. Conclusions

Salivary biomarkers, such as cortisol, salivary α-amylase, immunoglobulins, and others, could be appropriate for children as they are painless to collect. However, children in different stages of development and with various health conditions could have different hormonal and protein levels, with no clear predetermined salivary levels. Thus, the field of biomarkers in pain diagnostics is still being explored.

## Figures and Tables

**Table 1:** Filters that were applied after the initial search.

Date range:	within ten years (until 1 February 2023)
Species:	Human
Language:	English

**Table 2:** Criteria for inclusion and exclusion into our study.

Inclusion	Exclusion
Free text (also an external link to other sites)	Chronic pain, fibromyalgia, cancer-related pain, chronic pain exacerbation, pain management only
Salivary or sweat biomarkers	Serum, plasma, and urine biomarkers
Original research	Routinely used biomarkers in pain assessment, such as physiological parameters, pain scales, etc.
	In vitro and animal studies
	Literature reviews
	Systematic reviews, meta-analyses
	Abstract only with no external link
	Older than 10 years

**Table 3:** The main findings from the articles are included in the analysis.

Pediatric Studies									
No	Author	Date	Country	Age (Years) ± SD or IQR	Study Type	Sample Size	Groups	Biomarker/Biomarkers	Summary
1.	S. Brummelte et al. [24]	2015	Canada	At the age of 7 y (previously preterm)	Longitudinal cohort	156 children and their caregivers (98% mothers)	Three groups: ELGA, VLGA, term	Salivary cortisol	Three saliva samples were collected: pre-test, 30 min after the test at the end of the visit. The number of neonatal invasive procedures was associated with reduced white and grey matter development underlining the vulnerability of the brain at this early stage.
2.	G. Brockington et al. [25]	2021	Sweden	7.20 ± 2.12 y	Double-blinded experimental	81 children hospitalized in ICU	Two groups: storytelling *n* = 41; riddle *n* = 40	Salivary OXT; cortisol	The salivary sample was collected around 3:00 pm and was measured pre- and post-intervention (riddle or storytelling). A combination of increased OXT and reduced cortisol was observed to reduce the negative emotions associated with hospitalization and influence children’s perception of pain.
3.	N.C. Bavbek et al. [26]	2021	Germany	14.57 ± 2.39 y	Longitudinal prospective	18; 8 male, 10 female	-	sAA, sIgA and CgA; VAS pain score; pain catastrophizing scale; Corah’s dental anxiety scale	Salivary samples were collected between 8:30 and 10:00 am pre-procedure, 1 h after activation, on days 4, 7, 14, and 30 of retraction. The stress of starting orthodontic treatment increased sAA levels more compared to pain levels. Being male was a predictor of higher sAA concentrations.
4.	B.N. Jenkins et al. [27]	2018	USA	11.67 ± 3.79 y	Experimental pain procedure	73 tumor patients; 62% male	3 different emotion regulation conditions (distraction, reappraisal, reassurance)	sAA	Samples were collected 15 min before, immediately after, and 15 min after the procedure. sAA increased immediately after, but not during stress.
5.	F.J. Symons et al. [28]	2015	USA	9.2 ± 5.3 y	Not identified	10 children with CP; 8 male	-	Salivary cytokines/chemokines (IL-1α, IL8, MCP1), AgRP, prolactin, cortisol, DA, neuropeptide Y, somatostatin, NGF; Dalhousie pain interview (parental report)	Saliva was collected between 7:00–9:00 am. The interleukins (IL-1α, IL8) indicate the probability of an ongoing upregulation of the immune system among individuals with ongoing pain.
6.	M. Filippa et al. [29]	2021	Italy	10 days of age (premature)	Case-control	21, preterm infants in NICU; 45% female	-	Salivary oxytocin; PIPP-R pain score	In total, all included infants underwent a median of 7 painful procedures (3–15). Samples were collected before, 5, and 10 min after the heel stick procedure. The study revealed thatoxytocin system plays a crucial role in repairing and reconstructing the infant’s resilience to painful stimuli.
7.	M.J. da Silva Campos et al. [30]	2015	Poland	11–13 y	double-blinded experimental	20	-	sIgA	A negative correlation between oral pain intensity and salivary sIgA levels in children may indicate the importance of sIgA for oral protection during orthodontic treatment interfering with the pain experienced by the patients.
8.	D.Q. Stoye et al. [31]	2022	UK	4months chronological age	Case-control	58	42 very preterm, 16 extremely preterm infants	Salivary cortisol	Salivary cortisol was measured in prevaccination and postvaccination, in the morning and evening. Cortisol levels were higher in the preterm infants at 30 min post-stressor but not for the pre-stressor or 20-min post-stressor. Very premature infants showed decreased cortisol levels in response to vaccination.
9.	M. Carlton et al. [32]	2022	Australia	2–16 y	Exploratory cohort	20 children with burns	-	IL-1β, IL-4	Salivary cytokine levels were measured in burnt and healthy children. IL-and 1β, IL-4 concentrations were higher in the burnt group compared to the healthy controls.
10.	L. Marques-Feixa et al. [33]	2022	Spain	7–17 y	Case–control	54 with a current psychiatric diagnostic	-	sIgA	Saliva was collected during an acute laboratory-based psychosocial stressor. Although sIgA levels increased in adolescents, children showed no sIgA response to stress.
**Adult studies**									
**No**	**Author**	**Date**	**Country**	**Age (years) ± SD or IQR**	**Study type**	**Sample size**	**Control size**	**Biomarker**	**Summary**
1.	B.R. Goodin et al. [34]	2012	USA	21 ± 2.5 y	Cohort	36 (47% female)	-	Salivary cortisol, sTNFαRII	Cortisol was collected upon awakening, 15, 30, and 60 min after awakening. there was no support to provide for the association of CAR with cortisol or sTNFαRII responses to acute pain.
2.	J.C. Choi et al. [35]	2012	Republic of Korea	26.2 (3.2) y	Experimental	46 healthy male	-	Salivary testosterone, cortisol; pain threshold measurement; anxiety rating, ABP, HR measurements	This study revealed that acute clinical pain may be relieved by managing stress and controlling consequent stress-related testosterone and cortisol levels.
3.	J.C. Choi et al. [36]	2014	Republic of Korea	26.20 ± 3.14 y	Experimental	46; 25 tested in the spring; 21 in the summer	-	Testosterone, cortisol; anxiety rating at rest and under examination	Cortisol levels, pain ratings, and anxiety ratings were significantly higher under anxiety than at rest.
4.	K.M. Godfrey et al. [37]	2017	USA	mean age 29 y	Experimental	99 female twin pairs (*n* = 198); 88 complete data; 77%—monozygous twins	-	Salivary cortisol; pain rating and tolerance	Salivary cortisol was collected at home twice a day for seven consecutive days. First collection at 30 min after waking up, second—30 min before going to bed or by 11:30 pm. Day 3—induction with dexamethasone. The HPA axis is related to pain sensitivity and endogenous pain inhibition in response to a negative feedback paradigm and in recovery periods.
5.	H. Sadi et al. [38]	2013	USA	19–76 y	Cross-sectional	46 (43% female)	-	Salivary cortisol; sAA; dental anxiety scale	Samples were collected at 9:00 am and noon. The study failed to show any significant correlation between dental anxiety, cortisol, and sAA levels.
8.	E.M. Sobas et al. [39]	2016	Spain	30–40 y	Case study	34, 11 male, 23 female	-	Cortisol, sAA, sIgA, testosterone, sTNFaRII	Samples were collected at 9:00 and 12:00 am. sIgA and sTNFαRII levels were reproducible in healthy participants.
9	T.F. Robles. et al. [40]	2012	USA	18–40 y	Prospective cohort	76	-	sAA; self-reports of pain and anxiety	sAA were collected before, during, and post-surgery. Greater pain ratings at the follow-up visit were related to elevated sAA values. Higher pain ratings were related to lower sAA. Pain ratings during the surgery visit were not associated with sAA levels, and greater pain levels during the follow-up visit were related to greater sAA values).
10.	T.F. Robles et al. [40]	2012	USA	24.6 ± 5.2 y	Prospective, cohort	76 healthy participants	-	sAA; pain catastrophizing scale	sAA was measured preoperation, during surgery, and post-surgery via biosensor. Portable stress measurement devices, such as the sAA biosensor may eventually result in better pain control in patients undergoing surgical procedures.
11.	N. Christidis et al. [41]	2020	Sweden	23.8 ± 2.6 y	Case study	26 healthy participants; 13 male and 13 female	-	sAA; anxiety and somatization questionnaires	Saliva samples were collected before and 3 and 15 min after the painful stimulus. Experimentally induced muscle pain did not influence sAA levels.
12.	D. Koh et al. [42]	2014	Singapore	22–61 y	Not identified	58 male; 33 re-examined in two weeks	-	sAA	sAA was samples were collected 15 min and 1 min before, 1 and 15 min after venipuncture. sAA increased in response to the acute stress and remained elevated 15 min after the procedure.
13.	E.M. Sobas et al. [43]	2020	Spain	28.78 ± 6.93 y	Multicenter, prospective, descriptive cohort study	32; 19 male, 13 female.	-	sIgA, sTNFαRII; anxiety, depression, and quality of life questionnaires; VAS and NPRS pain scores	Samples were collected at 4-time points: baseline, pre-surgery, 1 h, and 72 h post-surgery. The concentration of sIgA and sTNFαRII post-surgery was significantly higher at each visit compared to baseline.
14.	M.S. Ozdogan et al. [44]	2018	Turkey	37.23 ± 12.97 y	Case-control study	39	-	Salivary opiorphin; VAS pain score	The salivary sample was collected pre-treatment, 7 days, and 30 days post-treatment. Salivary opiorphin levels increased in inflammation-related dental pain. The level of salivary opiorphin was strongly correlated with the reported level of pain.
15.	S. Bialka et al. [45]	2021	Poland	18–75 y	Case-control study with randomization	37	Control group, ThPVB group	sAA, testosterone; cortisol; sIgA; β-endorphin; VAS pain score	Salivary markers were collected before surgery, 6 and 24 h post-surgery. There was a statistically significant correlation between the VAS pain scale and sAA.
16.	A. Skvortsovaet al. [46]	2020	Netherlands	21.6 y	Randomized single-blinded controlled trial	99 women (taking oral contraceptives); in total 1235 saliva samples	Conditioned group, control group, drug group	Salivary OXT	The conditioned group received oxytocin nasal spray. Neither endogenous conditioned oxytocin release nor exogenous oxytocin administration influenced pain sensitivity.
17.	K.C. Lee et al. [47]	2017	USA	41.26 ± 13.56 y	Cross-sectional study (pilot study)	41, in total 23 were included; 43.9% male	-	sAA, dental anxiety score (Norman Corah’s dental questionnaire)	Data were collected between 1–4 pm. Baseline sAA concentration and output rate were not related to the procedure.
18.	R. Singh et al. [48]	2019	India	18–24 y	Case-control study	16; 6 male, 10 female	three groups according to orthodontic force (50–150 g)	IL-1β	A continuous force of 150 g resulted in significantly higher IL-1β levels at 24 h and after 2 months of initial canine tooth movement compared to control teeth.
19.	H. Jasim et al. [49]	2018	Sweden	24.8 ± 3.1 y	Experimental	20 healthy participants	-	Salivary NGF, CGRP, BDNF, sP; patient health questionnaire, perceived stress scale-10, generalized anxiety disorder scale, etc.	Saliva was collected at the same time between 8:30–10:30 am. A new protocol/method for NGF, CGRP, BDNF, and sP detection has been developed. Several isoforms of NGF, CGRP, and BDNF in human saliva have been detected.
20.	H. Jasim et al. [50]	2019	Sweden	26.3 ± 3.1	Experimental	10		Salivary NGF; BDNF, sP, glutamate	Samples were collected at 7:30, 10:30, 13:30, 16:30, and 19:30. The highest concentrations of the neuropeptides were expressed in the early morning, and they decreased across the day. The expression of salivary glutamate and sP did not show any significant changes across the day. NGF and BDNF in the whole saliva showed a significant variation across the day, but no variation in the levels of sP and glutamate was detected.

SD—standard deviation, IQR—interquartile range, ICU—intensive care unit, NICU—neonatal intensive care unit, AM—ante meridiem, PM—post meridiem, OXT—oxytocin, sAA—salivary alpha-amylase, HPA—hypothalamic-pituitary-adrenal, sIgA—salivary immunoglobulin A, sTNFαRII—soluble Tumor Necrosis Factor-α Receptor Type II, min—minutes, h—hour, g—gram, n—number, y—year, NGF—nerve growth factor, BDNF—brain-derived neurotrophic factor, sP—substance P, CGRP—calcitonin gene-related peptide, Il—interleukin, Il-1b—interleukin-1beta, CP—cerebral palsy, ELGA—extremely low gestational age, VLGA—very low gestational age, VAS pain score—Visual Analogue Scale, NPRS—Numeric Pain rating scale, MCP1—monocyte chemoattractant protein-1, AgRP—agoutine related peptide, CAR—cortisol awakening response, ABP—arterial blood pressure, HR—heart rate, ThPVB—thoracic paravertebral block, USA—United States of America, UK—United Kingdom, AM—ante meridiem, PM—post meridiem.

## Data Availability

All the data is available under request.
